# Supplementary material for: High SOX9 Maintains Glioma Stem Cell Activity through a Regulatory Loop Involving STAT3 and PML
Source: Int J Mol Sci. 2022 Apr 19;23(9):4511. doi: 10.3390/ijms23094511 (PMC9104987; doi:10.3390/ijms23094511)
Supplement: Supplementary file 1 [file ijms-23-04511-s001.zip › ijms-1655409-supplementary.pdf]

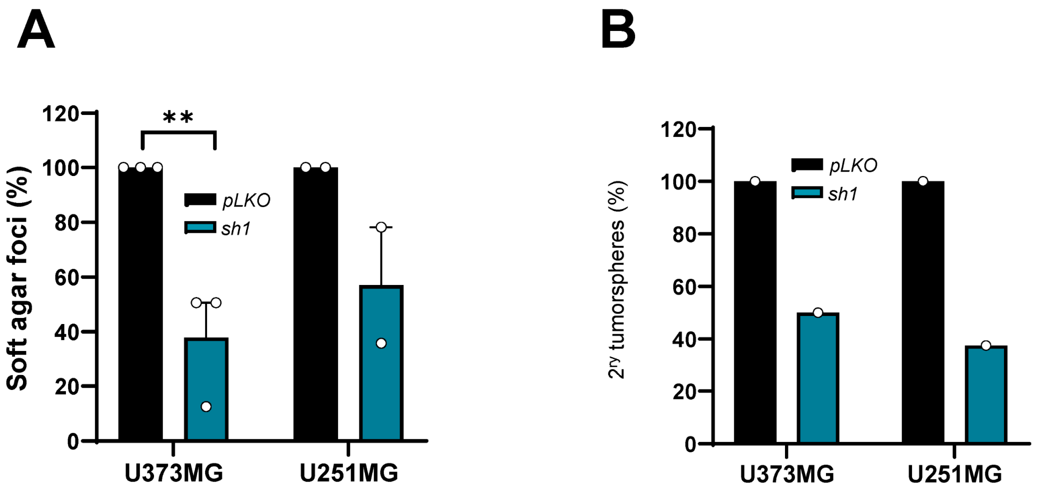

Figure S1. SOX9 knock-down impairs tumorigenicity. (A) Quantification of number of foci formed in soft agar from glioma cell lines infected with pLKO or sh1 plasmids (n=2). (B) Quantification of secondary tumorspheres after 10 days in culture post disgreggation of primary tumorspheres.

Supplemental Table S1. List of genes differentially expressed between *pLKO* and *shSOX9* GNS166 cells.

ID	control Avg (log2)	shRNA Avg (log2)	Fold change	P-val	FDR P-val	Gene Symbol	Description
202936_s_at	10.28	8.68	3.03	3.88E-07	0.0212	SOX9	SRY box 9
1555564_a_at	7.52	6.13	2.62	1.48E-06	0.0405	CFI	complement factor I
202935_s_at	9.82	8.79	2.05	4.67E-06	0.0474	SOX9	SRY box 9
225328_at	11.39	10.35	2.06	6.41E-06	0.0474	FBXO32	F-box protein 32
205698_s_at	8.48	7.3	2.27	6.61E-06	0.0474	MAP2K6	mitogen-activated protein kinase kinase 6
202206_at	10.55	9.33	2.32	7.46E-06	0.0474	ARL4C	ADP-ribosylation factor like GTPase 4C
207010_at	4.92	6.28	-2.57	1.18E-05	0.0474	GABRB1	gamma-aminobutyric acid (GABA) A receptor, beta 1
232195_at	7.01	8.17	-2.23	1.23E-05	0.0474	GPR158	G protein-coupled receptor 158
203854_at	9.49	8.44	2.08	1.49E-05	0.0480	CFI	complement factor I
1570469_at	7.33	6.19	2.21	1.68E-05	0.0510		
219857_at	6.83	5.77	2.09	2.50E-05	0.0538	PLEKHS1	pleckstrin homology domain containing, family S member 1
203963_at	9.25	8.23	2.03	2.56E-05	0.0538	CA12	carbonic anhydrase XII
228501_at	7.52	6.35	2.24	2.79E-05	0.0538	GALNT15	polypeptide N-acetylgalactosaminyltransferase 15
242979_at	6.8	5.76	2.05	3.15E-05	0.0538	IRS1	insulin receptor substrate 1
228245_s_at	7.91	8.92	-2.02	3.30E-05	0.0546	LOC728715	ovostatin homolog 2
235276_at	9.02	7.81	2.32	3.45E-05	0.0555	EPSTI1	epithelial stromal interaction 1 (breast)
209309_at	4.75	5.92	-2.25	3.63E-05	0.0566	AZGP1	alpha-2-glycoprotein 1, zinc-binding
229947_at	10.17	8.83	2.52	4.00E-05	0.0575	PI15	peptidase inhibitor 15
213201_s_at	6.92	7.93	-2.01	5.27E-05	0.0599	TNNT1	troponin T type 1 (skeletal, slow)
212985_at	9.64	8.59	2.08	5.57E-05	0.0603	APBB2	amyloid beta (A4) precursor protein-binding, family B, member 2
1552754_a_at	5.89	7.18	-2.44	6.68E-05	0.0617	CADM2	cell adhesion molecule 2
218152_at	9.28	8.14	2.21	7.78E-05	0.0624	HMG20A	high mobility group 20A
210794_s_at	8.92	9.98	-2.09	8.13E-05	0.0625	MEG3	maternally expressed 3 (non-protein coding)
1569727_at	5.2	6.37	-2.25	9.67E-05	0.0660	LOC101927701	uncharacterized LOC101927701
209493_at	8.82	7.8	2.02	9.94E-05	0.0660	PDZD2	PDZ domain containing 2
222986_s_at	11.16	10.11	2.06	0.0001	0.0660	SHISA5	shisa family member 5

201601_x_at	13.46	11.53	3.81	0.0001	0.0660	IFITM1, IFITM2	interferon induced transmembrane protein 1 and 2
227439_at	7.1	9.01	-3.75	0.0001	0.0660	ANKS1B	ankyrin repeat and sterile alpha motif domain containing 1B
237105_at	6.33	5.14	2.28	0.0001	0.0660	LOC101927027	uncharacterized LOC101927027
235118_at	4.16	5.56	-2.64	0.0001	0.0660	CADM2	cell adhesion molecule 2
228873_at	8.1	9.12	-2.04	0.0001	0.0686	COL22A1	collagen, type XXII, alpha 1
205625_s_at	8.72	9.9	-2.27	0.0002	0.0721	CALB1	calbindin 1
225345_s_at	8.5	7.31	2.28	0.0002	0.0724	FBXO32	F-box protein 32
228439_at	7.85	6.64	2.33	0.0002	0.0728	BATF2	basic leucine zipper transcription factor, ATF-like 2
206683_at	6.27	7.41	-2.21	0.0002	0.0771	ZNF165	zinc finger protein 165
202411_at	11.56	10.43	2.18	0.0002	0.0790	IFI27	interferon, alpha-inducible protein 27
229927_at	8.78	7.44	2.55	0.0002	0.0790	LEMD1	LEM domain containing 1
213241_at	8.31	7.1	2.31	0.0002	0.0790	PLXNC1	plexin C1
228367_at	9.02	7.94	2.13	0.0003	0.0793	ALPK2	alpha kinase 2
213421_x_at	6.81	8.35	-2.91	0.0003	0.0848	PRSS3	protease, serine, 3
1555464_at	5.71	4.34	2.58	0.0003	0.0849	IFIH1	interferon induced, with helicase C domain 1
202508_s_at	7.25	8.26	-2.01	0.0004	0.0912	SNAP25	synaptosome associated protein 25kDa
200629_at	12.03	10.82	2.32	0.0004	0.0920	WARS	tryptophanyl-tRNA synthetase
201641_at	8.8	7.76	2.06	0.0004	0.0934	BST2	bone marrow stromal cell antigen 2
211478_s_at	4.93	6.02	-2.13	0.0004	0.0934	DPP4	dipeptidyl-peptidase 4
204994_at	10.15	8.68	2.77	0.0004	0.0934	MX2	MX dynamin-like GTPase 2
205623_at	10.1	9.01	2.12	0.0004	0.0962	ALDH3A1	aldehyde dehydrogenase 3 family, member A1
214022_s_at	13.17	11.54	3.09	0.0005	0.0965	IFITM1	interferon induced transmembrane protein 1
232355_at	6.71	7.73	-2.04	0.0005	0.0965	SNORD114-3	small nucleolar RNA, C/D box 114-3
209417_s_at	11.15	10.01	2.21	0.0005	0.0965	IFI35	interferon-induced protein 35
204972_at	9.66	8.49	2.26	0.0005	0.0990	OAS2	2'-5'-oligoadenylate synthetase 2
203717_at	7.15	8.22	-2.1	0.0005	0.1008	DPP4	dipeptidyl-peptidase 4
222793_at	9.73	8.24	2.81	0.0006	0.1045	DDX58	DEAD (Asp-Glu-Ala-Asp) box polypeptide 58
207463_x_at	6.64	8.04	-2.64	0.0006	0.1068	PRSS3	protease, serine, 3
226459_at	9.9	8.02	3.69	0.0006	0.1068	PIK3AP1	phosphoinositide-3-kinase adaptor protein 1
202687_s_at	9.67	8.43	2.36	0.0006	0.1068	TNFSF10	tumor necrosis factor (ligand) superfamily, member 10
204439_at	12.36	11.09	2.41	0.0008	0.1113	IFI44L	interferon-induced protein 44-like
218943_s_at	9.7	8.24	2.75	0.0008	0.1113	DDX58	DEAD (Asp-Glu-Ala-Asp) box polypeptide 58
200628_s_at	10.28	9.11	2.24	0.0008	0.1144	WARS	tryptophanyl-tRNA synthetase
205660_at	10.06	8.59	2.78	0.0009	0.1196	OASL	2'-5'-oligoadenylate synthetase-like
227441_s_at	3.39	4.52	-2.18	0.0010	0.1212	ANKS1B	ankyrin repeat and sterile alpha motif domain containing 1B
210797_s_at	9.65	8.26	2.63	0.0010	0.1221	OASL	2'-5'-oligoadenylate synthetase-like
227440_at	5.22	6.37	-2.22	0.0012	0.1324	ANKS1B	ankyrin repeat and sterile alpha motif domain containing 1B
240292_x_at	3.37	4.82	-2.75	0.0014	0.1423	ANKS1B	ankyrin repeat and sterile alpha motif domain containing 1B
235489_at	7.1	6.1	2	0.0015	0.1438	RHOJ	ras homolog family member J
202269_x_at	10.62	9.52	2.14	0.0016	0.1471	GBP1	guanylate binding protein 1, interferon-inducible
202688_at	9.58	8.47	2.16	0.0016	0.1474	TNFSF10	tumor necrosis factor (ligand) superfamily, member 10
205552_s_at	10.14	9.09	2.07	0.0018	0.1549	OAS1	2'-5'-oligoadenylate synthetase 1
204187_at	9.51	8.42	2.12	0.0019	0.1558	GMPR	guanosine monophosphate reductase
213797_at	9.9	8.35	2.93	0.0019	0.1558	RSAD2	radical S-adenosyl methionine domain containing 2

242625_at	10.37	8.87	2.83	0.0019	0.1576	RSAD2	radical S-adenosyl methionine domain containing 2
204533_at	9.25	7.15	4.3	0.0021	0.1629	CXCL10	chemokine (C-X-C motif) ligand 10
228152_s_at	10.88	9.57	2.48	0.0021	0.1631	DDX60L	DEAD (Asp-Glu-Ala-Asp) box polypeptide 60-like
205699_at	6.58	5.49	2.12	0.0022	0.1654	MAP2K6	mitogen-activated protein kinase kinase 6
230865_at	6.62	5.34	2.43	0.0022	0.1654	LIX1	limb and CNS expressed 1
210163_at	6.54	5.32	2.33	0.0022	0.1655	CXCL11	chemokine (C-X-C motif) ligand 11
223502_s_at	9.25	7.23	4.04	0.0022	0.1657	TNFSF13B	tumor necrosis factor (ligand) superfamily, member 13b
229764_at	7.78	6.61	2.26	0.0024	0.1680	TPRG1	tumor protein p63 regulated 1
208025_s_at	5.99	7.12	-2.18	0.0026	0.1748	HMGA2	high mobility group AT-hook 2
1558945_s_at	4	5.19	-2.27	0.0027	0.1778	CACNA1A	calcium channel, voltage-dependent, P/Q type, alpha 1A subunit
231577_s_at	10.92	9.81	2.16	0.0028	0.1794	GBP1	guanylate binding protein 1, interferon-inducible
241014_at	9.14	8.1	2.06	0.0028	0.1794	FLG-AS1	FLG antisense RNA 1
202311_s_at	7.77	6.64	2.19	0.0028	0.1794	COL1A1	collagen, type I, alpha 1
228329_at	6.13	7.19	-2.08	0.0028	0.1794	DAB1	Dab, reelin signal transducer, homolog 1 (Drosophila)
205498_at	4.67	5.69	-2.02	0.0030	0.1839	GHR	growth hormone receptor
223501_at	8.92	6.95	3.93	0.0043	0.2037	TNFSF13B	tumor necrosis factor (ligand) superfamily, member 13b
230650_at	6.35	7.57	-2.33	0.0043	0.2043	SLCO5A1	solute carrier organic anion transporter family, member 5A1
226702_at	11.31	10.17	2.21	0.0046	0.2099	CMPK2	cytidine monophosphate (UMP-CMP) kinase 2, mitochondrial
211122_s_at	7.51	6.32	2.29	0.0047	0.2113	CXCL11	chemokine (C-X-C motif) ligand 11
230383_x_at	7.73	6.67	2.09	0.0050	0.2189	SLFN5	schlafen family member 5
223614_at	7.44	8.52	-2.11	0.0050	0.2189	MMP16	matrix metallopeptidase 16 (membrane-inserted)
222802_at	8.58	7.57	2.02	0.0069	0.2461	EDN1	endothelin 1
242961_x_at	9.69	8.54	2.23	0.0117	0.3033	DDX58	DEAD (Asp-Glu-Ala-Asp) box polypeptide 58
210785_s_at	8.88	7.86	2.03	0.0378	0.4571	THEMIS2	thymocyte selection associated family member 2
